# The exploration of the dispersal of British military families in England following the Strategic Defence and Security Review 2010

**DOI:** 10.1371/journal.pone.0238508

**Published:** 2020-09-08

**Authors:** Michael Rodrigues, Alison K. Osborne, Derek Johnson, Matthew D. Kiernan

**Affiliations:** 1 Faculty of Health and Life Sciences, Northumbria University, Newcastle upon Tyne, United Kingdom; 2 Faculty of Engineering and Environment, Northumbria University, Newcastle upon Tyne, United Kingdom; US Army Research Institute of Environmental Medicine, UNITED STATES

## Abstract

Strictly relying on publicly available data, this study depicts and quantifies the spatial pattern of England’s military families with dependent children. England’s Service Pupil Premium for the financial years between 2011 and 2019 is used as a proxy variable to estimate the density of service children at the parliamentary constituency level. Methodologically, the approach allows an assessment of spatial movements of a population or a cohort. The results inform policy makers by providing evidence-based findings about the location of England’s military families and how the distribution has changed between 2011 and 2019. The results show empirical evidence supporting the hypothesis that, at a macro scale, beyond commuting distance, England’s military families are becoming increasingly dispersed. We argue that the findings unveil spatial dynamics that have practical issues of housing, employment, and education regarding military families.

## 1. Introduction

Clever and Segal [1] argue that military families are a strikingly diverse population with diverse needs. Specifically, Tipping [2] has identified the social sacrifices military personnel and their families make upon joining the armed forces, including how this broaches wide areas of societal provision such as the education of children, approach to veterans, formal recognition and care of military families.

International research has predominantly focused on the impact of operational deployments on spouses and children of military service members [3–5]. US studies demonstrate that children of service personnel psychologically suffer when parents or siblings are deployed and with family integration upon return [6, 7]. Cliffton [8] found that, in the United Kingdom (UK), the service wife was pivotal to the whole family, if the mother was able to cope, then the likelihood was that the children would also adapt well [6, 9]. However, this is dependent on the psychological wellbeing of the non-serving parent. In the Army Families Federation (AFF) Command Brief, 79% of spouses stated that they had compromised on the wellbeing and mental health of the non-serving family members to varying degrees [10]. Research has suggested that social support often mitigates the psychological effects of military-induced separations [11–13].

In the UK, support for military families is predominantly focused on large military bases or garrisons. Organisations such as HIVE—Forces Information Centre support the service community through an information network on relocation, civilian facilities, healthcare access to name but a few as well as supporting families when serving partners are away on military operations. Organisations such as HIVE rely on their physical presence within a military base or garrison to engage the military families. Garrison or military base focused family support is generally not available beyond those boundaries and the further away the family is geographically, the less support it is able to access. The psychological impact of military life is potentially exacerbated when families live away from military communities where most of the support is available.

Publications from military charities suggest that military families are increasingly prioritising educational stability for their children, the employment of the non-serving partner and family support, as a reason for military families living away from military communities [10, 14]. However, these families risk a loss of connection with the wider military community, can develop a sense of vulnerability and experience poor communication with the serving family members unit [14].

The greatest challenge faced by those organisations providing support is the paucity of data on the geographical location of military families in the UK. A study carried out by the UK Ministry of Defence (UKMOD) reported that over 65,000 British regular trained military personnel were married or in a civil partnership [15]. However, this estimate was derived from a self-reported, non-compulsory data field on the UKMOD Joint Personnel Administration system, and did not include any data on the geospatial distribution of the families. When considering military families with school aged children, the situation becomes ever more challenging, as there is no public record of their numbers or locations in the UK.

The spatial distribution of UK military families has gained importance since the publication of the 2010 Strategic Defence and Security Review (SDSR) of the British Armed Forces [16]. Produced in October 2010, the SDSR was the first substantial UK defence and security policy review since 1998 and provided the strategic vision for the British Armed Forces in the upcoming years. The review set out the future structure of the UK military organisation, reduction in service manpower and included the withdrawal of all military personnel and families from UK bases in Germany by 2020. The withdrawal from Germany affected 20,000 serving personnel and their families and was a significant alteration to the service family’s geospatial distribution within the UK. The review also included the closure of smaller military installations within the UK and a move towards larger garrison communities such as Catterick Garrison in North Yorkshire and Tidworth Garrison Salisbury in the West of England.

Recent studies on UK military families have predominantly focused on wellbeing or psychological health [17, 18], but none have explored the geospatial distribution of military families. In addition, these studies have predominantly relied upon survey methods. When considering the scale of change to the military families geospatial picture and the need to understand how that has changed post SDSR at the macro (country) scale, such methodological approaches become problematic due to cost and time constraints [19].

In 2019, the UK House of Commons Defence Committee [20] raised concerns that the UK Ministry of Defence was not adapting support mechanisms to accommodate families that were living at considerable distances from the family’s serving member’s parent unit. The committee stipulated that family’s that were dispersed from the garrison or military base locations must not be disadvantaged in their access to support services. To support military families more effectively, the Defence Committee determined a need for quantitative evidence of family locations as this was central to the success of the UKMOD Future Accommodation Model (FAM) to succeed. FAM is a new service families accommodation initiative moving away from government provided service quarters or accommodation. It gives service personnel choices over where they live and who they live with, whilst still providing a monetary allowance for a family home [21]. It is hoped that this new model will improve family stability, however, the UKMOD accepts that it will lead to a wider dispersal of military families across the UK away from military bases and garrisons. As previously mentioned, and raised as a concern within the Defence Committee, current statutory data on family’s locations is poor and incomplete.

It is not uncommon for a government organisation not to have a direct measure of a particular phenomenon and in such cases it is widely accepted that reliable proxy data can bridge the information gap. A proxy measurement is an indirect measure of a desired outcome which is itself strongly correlated to that outcome [22]. Military families are at the core of the military community, therefore, knowing their location is of utmost importance for the future planning of support services.

In this study, the lack of centralised information on location and geospatial distribution of military families indicated the need to use proxy data. Proxy data is used by UK government to determine issues such as child poverty [23] and internationally as a socio-economic indicator [24, 25]. Therefore, in response to the UK governments need for a more accurate picture of the geographical location of military families, the aim of this exploratory study was to use a publicly available proxy measure to map the geospatial distribution of military families in England post SDSR 2010.

## 2. Methods

The availability of a constant proxy which correlated well with military families was very limited. For the purposes of this study, the family is defined as a family unit which has dependent children, and England’s Service Child Pupil Premium (SCPP) was used as the proxy measure. The SCPP program consists of funding for state schools, academies, and free schools in England to provide additional support to pupils from military families, acknowledging the specific challenges they may face [26]. SCPP was first introduced in April 2011 for service children aged 5–16. It is accepted that the data does not include those in private or boarding schools and children of reservists or full-time reservists on home commitment. However, it is the only proxy measure that identifies the majority of military families with children in England. SCPP data provides a spatial reference, enhancing the ability to spatially consider service children at the macro scale.

Data is made available at two aggregation levels: 1) local authority and 2) parliamentary constituency. This study used the constituencies as it is the most disaggregated level available. UK parliamentary constituency is a widely used unit of analysis of socio-economic and demographic data as they each contain roughly the same number of people [27]. The UK is currently divided into 650 parliamentary constituencies, each represented by one Member of Parliament in the House of Commons, 533 constituencies in England, 59 in Scotland, 40 in Wales, and 18 in Northern Ireland. Despite containing approximately the same number of people, the constituencies’ spatial extent differs significantly, England’s average being 675 km^2^ (*SD* ±1,064 km^2^). When considering the UK, the average area increases to 1,118 km^2^ (*SD* ±2,893 km^2^). Constituency boundaries have not undergone any changes over the study period.

Due to statistical disclosure control, constituencies with 1 to 4 registered service pupils are not disclosed in the publicly available data. Where no children are registered, a value of zero is present. In order to use all 533 geographical units in England a value denoting 2 pupils was assigned to all constituencies where the exact value is not revealed but known to be between 1 and 4 children. The approach to impute the missing values in the datasets was to replace the missing values with a constant numeric value [28]. A constant imputation approach replaces the missing values with any constant value depending upon the magnitudes of the individual attributes [28]. In the case of this study the missing values were known to range between 1 and 4. Therefore a decision was made to replace the missing values with a constant numeric value of 2. As can be expected, SCPP annual data is significantly skewed, recording similar high skewness values each year. Table 1 provides one example for the period 2016/17. This skewness arises because SCPP is for the most part concentrated in a small number of constituencies. To gain an estimated density of service children across England, the number of SCPP pupils were divided by the surface area of the constituency in question, expressed in square kilometres.

**Table 1:** SCPP descriptive statistics (2016/17).

**Mean**	141.04
**Standard Error**	11.57
**Median**	55.00
**Mode**	2.00
**Standard Deviation**	266.80
**Sample Variance**	71183.96
**Kurtosis**	33.09
**Skewness**	4.91
**Range**	2785.00
**Maximum**	2785.00
**Minimum**	0.00
**Sum**	75033.00

### 2.1. Proximity analysis

Selecting a data subset of the constituencies above the mean for each financial year provided a straightforward spatio-temporal comparison of the data. The selected method was a simplified approach to nearest neighbour analysis [29], measuring the average distance between centroids of each constituency and its immediate neighbour. For each year, the subset of constituencies that were above England’s mean density of SCPP pupils were selected for analysis. With this criterion, if the number of SCPP pupils increases and average distance increases, greater distances are required to find constituencies with a density above the mean: i.e. evidence of concentration of military families at a macro scale. Conversely, if the number of SCPP pupils increases and the average distance decreases, a shorter distance is required: i.e. evidence of scattering of military families at a macro scale.

### 2.2. Global index of spatial autocorrelation

The second method applied allowed the assessment of spatial dependence of the density of SCPP pupils. Global Moran’s I, a global index of spatial autocorrelation, was used to test if constituencies with a similar density of SCPP pupils were located close together or if they were randomly distributed across England. There are a multitude of approaches to Global Moran’s I [30, 31, 32]. To ensure a consistent analysis with the large variation in constituencies’ size, and to assess the clustering of military families at shorter and longer distances, a decision was made to test for the presence of spatial autocorrelation at a range of band distances.

Spatial statistics need to consider the relations of proximity among geographical features prior to analysis. These spatial conceptualisations determine the spatial association and scale factors inherent in geographical research. A marked characteristic of any military family is their intertwined dependence with the military facility where the serving parent(s) is (are) stationed. There are three possibilities: 1) The serving parent(s) is(are) stationed and lives in the same constituency, as a result children attend a school in that same constituency, therefore the military family location is determined by the existence of a military facility; 2) The serving parent(s) is(are) commuting to another constituency on a daily basis, therefore their children attend school in a different constituency, however location is still ultimately determined by the existence of a military facility in a neighbouring constituency. 3) The serving parent(s) is(are) stationed outside of commuting distance and living geographically separated from the children. In this case the location of the military family, proxied by the children, is independent from the existence of a military facility, i.e. the location criterion is aspatial from a military facility.

Therefore, the main spatial conceptualisation adopted considered a fixed distance band to determine the constituencies’ neighbourhood, the aim being a simplified model of spatial interactions to test the short/long-distance rationale.

Each constituency was analysed within the context of neighbouring constituencies located within the distance band specified. Constituencies outside the specified distance did not influence calculations. This spatial conceptualisation was also appropriate for the analysis due to the large variation in the constituencies’ polygon size, ($\bar{\text{x}}=675$ km^2^; *SD* ±1,064 km^2^). For all measurements the bands used row-standardised weights and the distance method was Euclidean. Row standardisation was used to account for the aggregation scheme of the constituencies. This scaled all weights creating a relative, rather than absolute, weighting scheme [33] and allowed mitigation of bias due to constituencies having different numbers of neighbours.

The testing of incremental spatial autocorrelation identifies an appropriate distance band for which spatial autocorrelation is more pronounced [34]. To test for the appropriate distances, Global Moran’s I was run with increments of 15 km. A distance of 15 km was selected as the radius of the average constituency area (675 km^2^). Calculating Global Moran’s I with several distance bands evaluates whether a variable is clustered or dispersed across varying distances, suggesting different spatial relationships [35]. Therefore, the results of this method will show the concentration or dispersal of the military families’ community post SDSR 2010 in relation to each constituency. The method will also show any differences in the pattern when considering a shorter-distance and a macro scale.

### 2.3. Local indicator of spatial association

To find the main areas of concentration of military families with dependent children a local indicator of spatial association was used. Local spatial autocorrelation techniques, such as Getis G index [36] and Anselin local Moran’s I index [37] pinpoint hotspots and spatial clusters across the territory. The selected local indicator of spatial association for this research was Anselin local Moran’s I [37] which identifies spatial clusters according to the intensity of the variable across the neighbouring areas. In this study spatial clusters represent constituencies sharing a similarity or dissimilarly of values in the density of SCPP pupils. Thus, the method can show areas of identified concentration of military families that should be prioritised for interventions focusing on this cohort.

## 3. Results

**Fig 1:**
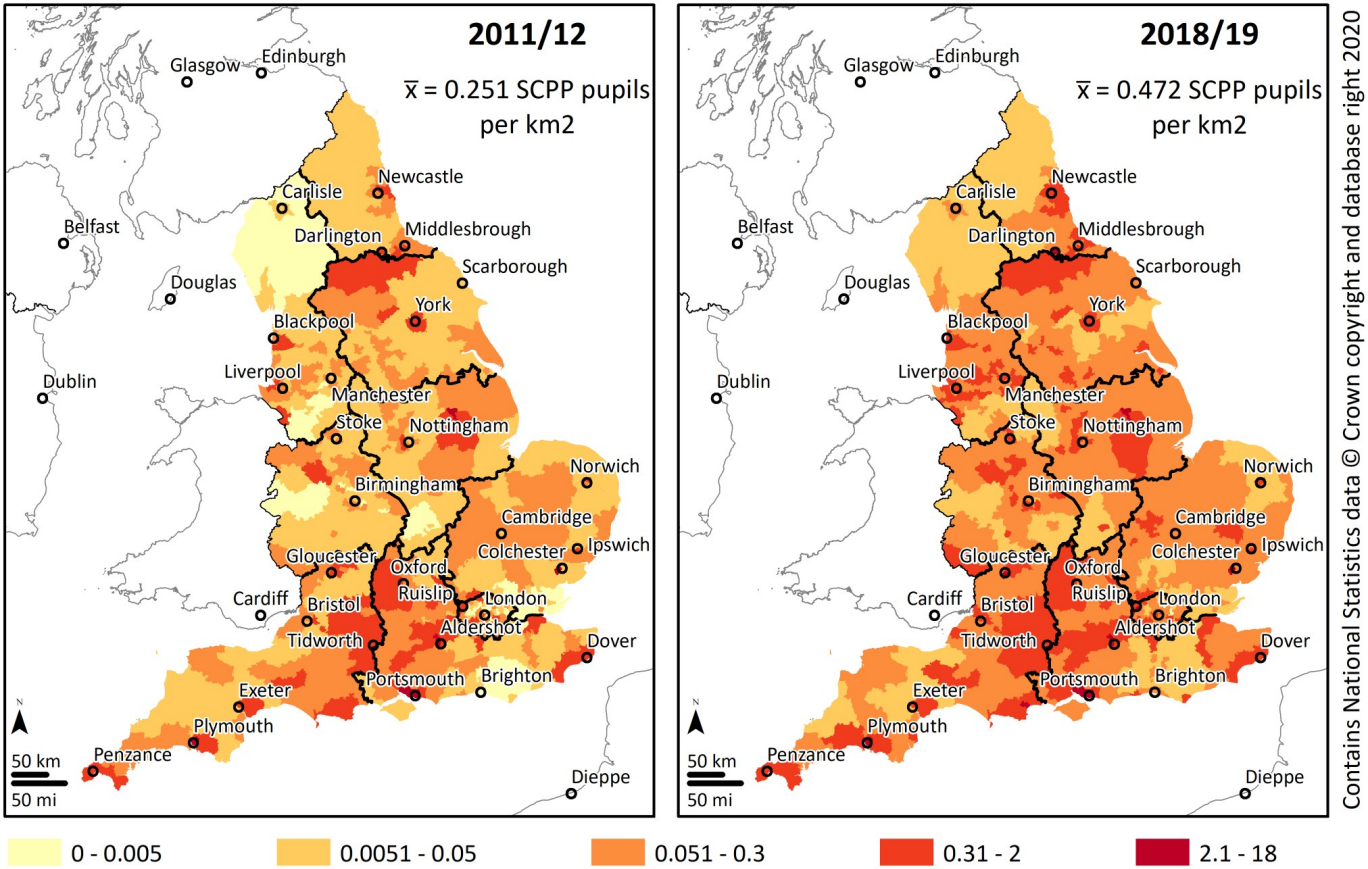
Density of SCPP pupils in England.

Contrary to the general population, military families do not tend to live in metropolitan areas. This is expected since many major military facilities, such as Catterick Garrison in North Yorkshire and Tidworth Garrison near Salisbury Palin, are in non-metropolitan areas. Fig 1 shows the general distribution of the SCPP pupils in the beginning and in the end of the study period. Fig 1 shows that the overall spatial pattern has not changed significantly over the study period, meaning that the areas of highest and lowest density of military families remained the same between 2011–2019. However, the data demonstrates that there has been a significant increase in the number of SCPP pupils during the period observed.

Table 2 shows the year on year increase in the count and density per km^2^. In the study period, following the implementation of SDSR 2010, the number of service children within England increased by 69% from 45,042 to 75,970 SCPP pupils. Consequently, between 2011 and 2019, England’s average density of SCPP pupils increased by 88%, from 0.251 service children per km^2^ at the start of the study period to 0.472 service children per km^2^ by 2018/19. It is possible to distinguish two phases which divide the study period, the first half, where the increase in service children was more noticeable, and the second half, where the increase has slowed and became more moderate. During 2011–2015 England’s service children increased by 44%, however, between 2016 and 2019 the increase was a more modest 11%.

**Table 2:** SCPP pupils in England at the constituency level.

	SCPP pupils (count)							Density per km^2^					
Financial year	N	% change	Maximum	Mean	Median	SD	Median/Mean ratio	Maximum	Mean	% change	Median	SD	Median/Mean ratio
2011/12	45,042	---	2046	85	15	204	0.176	14.382	0.251	---	0.051	0.995	0.203
2012/13	52,283	16%	2279	98	23	221	0.235	15.816	0.299	19%	0.083	1.089	0.278
2013/14	58,132	11%	2440	109	30	234	0.275	16.950	0.344	15%	0.118	1.162	0.343
2014/15	64,654	11%	2460	121	40	241	0.331	16.950	0.395	15%	0.164	1.199	0.415
2015/16	68,717	6%	2599	129	47	249	0.364	17.689	0.427	8%	0.187	1.263	0.438
2016/17	73,280	7%	2666	138	50	261	0.362	18.008	0.456	7%	0.208	1.298	0.456
2017/18	75,078	2%	2785	141	55	267	0.390	18.101	0.465	2%	0.210	1.319	0.452
2018/19	75,970	1%	2843	143	58	268	0.406	17.936	0.472	2%	0.205	1.316	0.434

**Fig 2:**
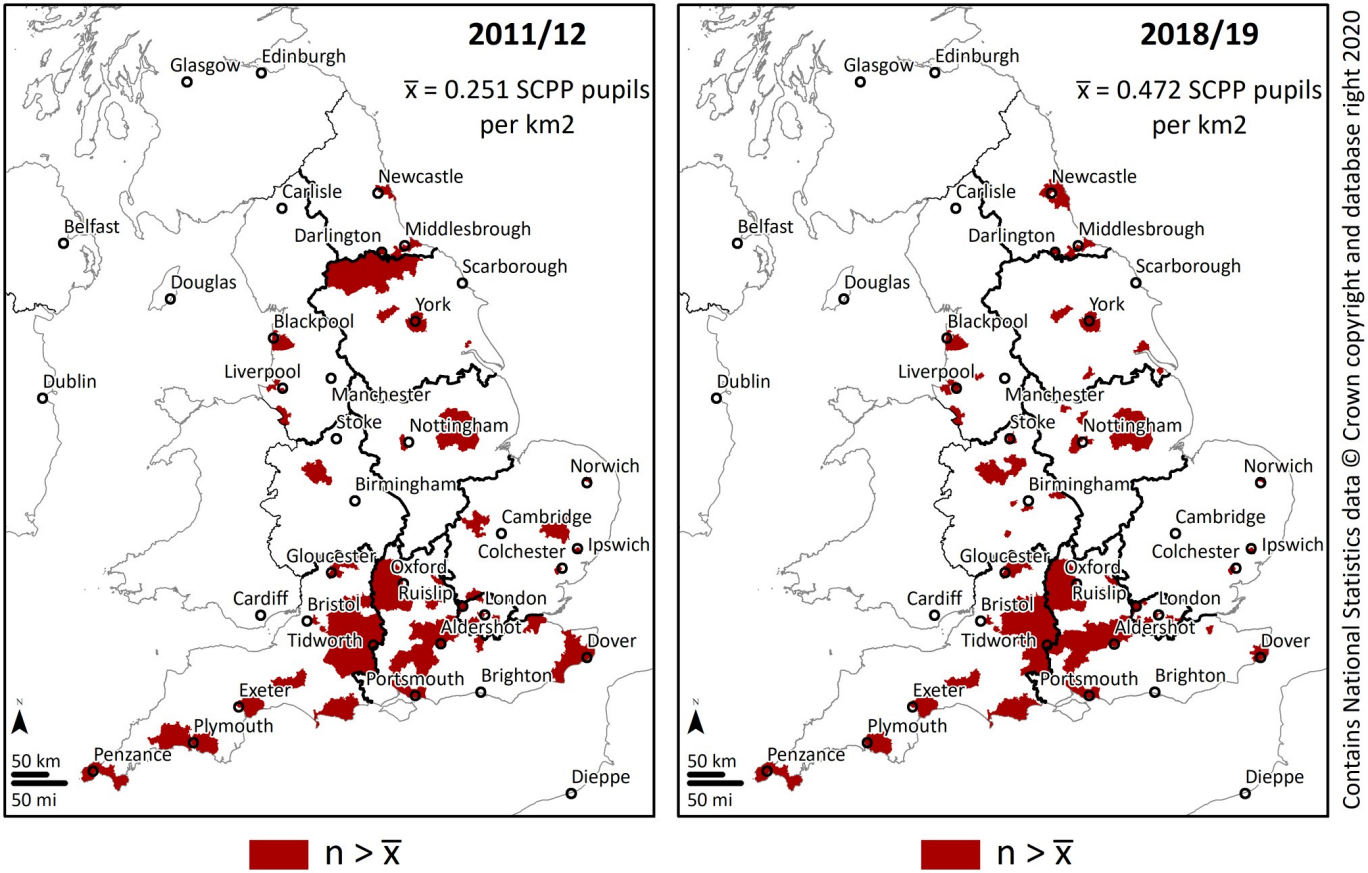
Constituencies with a density of SCPP pupils above England’s mean.

Overall, the results indicate an expected concentration of military families at very specific constituencies. In 2011/12, 83% (n = 443) of England’s 533 constituencies had a density of SCPP pupils below the mean, while in 2018/19 the amount decreased to 79% (n = 422). These initial results suggest that rather than concentrating in the same areas over the years, a small number of military families became progressively dispersed. Fig 2 shows locations of the constituencies with a density of SCPP pupils above England’s mean.

Noticeable is the increase of high-density constituencies in the central regions of England towards the end of the study period. Overall, the high-density core areas coincide with constituencies with a strong presence of military facilities. Close to Portsmouth, Gosport has HMS Sultan, the primary engineering training establishment for the Royal Navy. In Plymouth there is HMNB Devonport, the largest naval base in Western Europe. Tidworth, Aldershot and Colchester have significant British Army Garrisons. In Portsmouth, HMNB Portsmouth is headquarters for two-thirds of the Royal Navy’s surface fleet. In Fareham next to Portsmouth there is HMS Collingwood the lead establishment of the Maritime Warfare School and the largest naval training organisation in Western Europe. Darlington and Middlesbrough are within commuting distance of Catterick Garrison, the largest of the British Army’s Garrisons. Gloucester is home to NATO’s Allied Rapid Reaction Corps which relocated to the UK from Germany in 2010.

### 3.1. Proximity analysis

Table 3 shows results from the average nearest neighbour analysis, showing that during the study period, constituencies with densities above the mean report a trend towards dispersion. In 2011/12 there were 90 constituencies with a density of SCPP pupils above England’s mean. The average distance among this subset of 90 polygons’ centroids was approximately 24 km. By the end of the study period the number increased to 111 constituencies (+23%). Moreover, the results show that the average distance among the subset of 111 polygons’ centroids was standing at approximately 21 km. Therefore by 2018/2019 one would need to travel a shorter distance to find a constituency with a high density of SCPP pupils—indicating families’ dispersal at the macro scale. This finding also suggests that, over the study period, an increasing number of military families has chosen to live beyond the high-density core areas, at greater distances from military garrisons and bases.

**Table 3:** Average nearest neighbour statistics.

Financial year	England’s density of SCPP pupils per km^2^	Constituencies sample subset	Constituencies sample subset: mean distance among centroids (km)
2011/12 N = 533	$\bar{\text{x}}=0.251$	$\text{n}>\bar{\text{x}}=90$	23.885
2012/13 N = 533	$\bar{\text{x}}=0.299$	$\text{n}>\bar{\text{x}}=97$	23.542
2013/14 N = 533	$\bar{\text{x}}=0.344$	$\text{n}>\bar{\text{x}}=101$	21.727
2014/15 N = 533	$\bar{\text{x}}=0.395$	$\text{n}>\bar{\text{x}}=113$	19.948
2015/16 N = 533	$\bar{\text{x}}=0.427$	$\text{n}>\bar{\text{x}}=113$	20.101
2016/17 N = 533	$\bar{\text{x}}=0.456$	$\text{n}>\bar{\text{x}}=112$	20.848
2017/18 N = 533	$\bar{\text{x}}=0.465$	$\text{n}>\bar{\text{x}}=119$	20.498
2018/19 N = 533	$\bar{\text{x}}=0.472$	$\text{n}>\bar{\text{x}}=111$	20.574

### 3.2. Global index of spatial autocorrelation

The density of SCPP pupils was tested for clustering in a radius between 15 and 450 km from each constituency. Table 4 shows where the resulting z-scores peak.

**Table 4:** Global Moran’s I summary by distance band.

Financial year	First Peak		Max Peak	
	Distance (km)	z-score	Distance (km)	z-score
2011/12	105	9.579	420	15.215
2012/13	105	9.592	420	15.008
2013/14	105	9.607	420	14.554
2014/15	105	9.583	420	14.076
2015/16	105	9.416	420	13.730
2016/17	105	9.260	420	13.201
2017/18	105	9.187	420	13.221
2018/19	105	9.650	420	13.239

Tested distance bands: 30; beginning distance: 15 km; distance increment: 15 km; Other parameters: row standardisation and Euclidian distance.

Results from Table 4 indicate a fixed distance band of 105 km (65 miles) as the first peak of clustering, while a distance band of 420 km (260 miles) reflects maximum spatial autocorrelation (i.e. maximum clustering for the density of SCPP pupils). The distance band that exhibits maximum clustering is the distance where the underlying spatial process is most pronounced across the landscape [38]. Therefore, the 105 km radius was considered as the reference for short distance. The 420 km radius, which reflects maximum spatial autocorrelation, was selected as the reference value for long-distance whilst also serving to indicate a macro threshold outside of commuting distance.

Following the confidence levels used in this study (95% confidence, alpha = 0.05), a positive Global Moran’s I and corresponding z-scores >1.96 indicate that military families are spatially concentrated through the clustering of similar values of the density of SCPP pupils. A negative Global Moran’s I and corresponding z-scores < −1.96 indicate that military families are spatially dispersed through the clustering of dissimilar values in the spatial distribution of the density of SCPP pupils.

Table 5 shows that all values of Global Moran’s I were positive, and z-scores passed through the 5% level of significance test. Therefore, confirming spatial autocorrelation and indicating that constituencies with similar densities of SCPP pupils tend to be adjacent across the territory. This demonstrates a spatially clustered pattern where constituencies with a high density of military families are close to each other, intercalated by constituencies with a lower density of military families.

**Table 5:** Global Moran’s I statistics.

	Distance band: 105 km			Distance band: 420 km		
Financial year	Moran’s Index	z-score	p-value	Moran’s Index	z-score	p-value
2011/12	0.0763	9.5789	0.000000	0.0227	15.2153	0.000000
2012/13	0.0765	9.5920	0.000000	0.0224	15.0084	0.000000
2013/14	0.0767	9.6069	0.000000	0.0217	14.5538	0.000000
2014/15	0.0773	9.5829	0.000000	0.0211	14.0755	0.000000
2015/16	0.0762	9.4155	0.000000	0.0206	13.7298	0.000000
2016/17	0.0751	9.2598	0.000000	0.0198	13.2013	0.000000
2017/18	0.0744	9.1875	0.000000	0.0198	13.2209	0.000000
2018/19	0.0786	9.6502	0.000000	0.0199	13.2388	0.000000

Distance method: Euclidian; Row standardisation.

Using the 105 km shorter distance band, where the first clustering peak occurs, Global Moran’s I increased between 2011 and 2015 (Table 5). Between 2015 and 2018 it decreased slightly before increasing again in 2018/19. Therefore, when considering a distance of 105 km relative to each constituency, the clustering of SCPP pupils has strengthened since 2011/12. Hence, when using a distance band of 105 km, military families tended to concentrate between the beginning and the end of the study period.

However, when increasing the distance of the analysis to 420 km, a threshold well beyond commuting distance, the results showed that 420 km was the distance where the underlying spatial processes are most pronounced and revealed maximum spatial autocorrelation. When considering a distance band of 420 km, Global Moran’s I decreased over the study period (Table 5). This finding suggests that, at a macro scale, beyond commuting distance, England’s military families become increasingly dispersed.

### 3.3. Local indicator of spatial association

**Fig 3:**
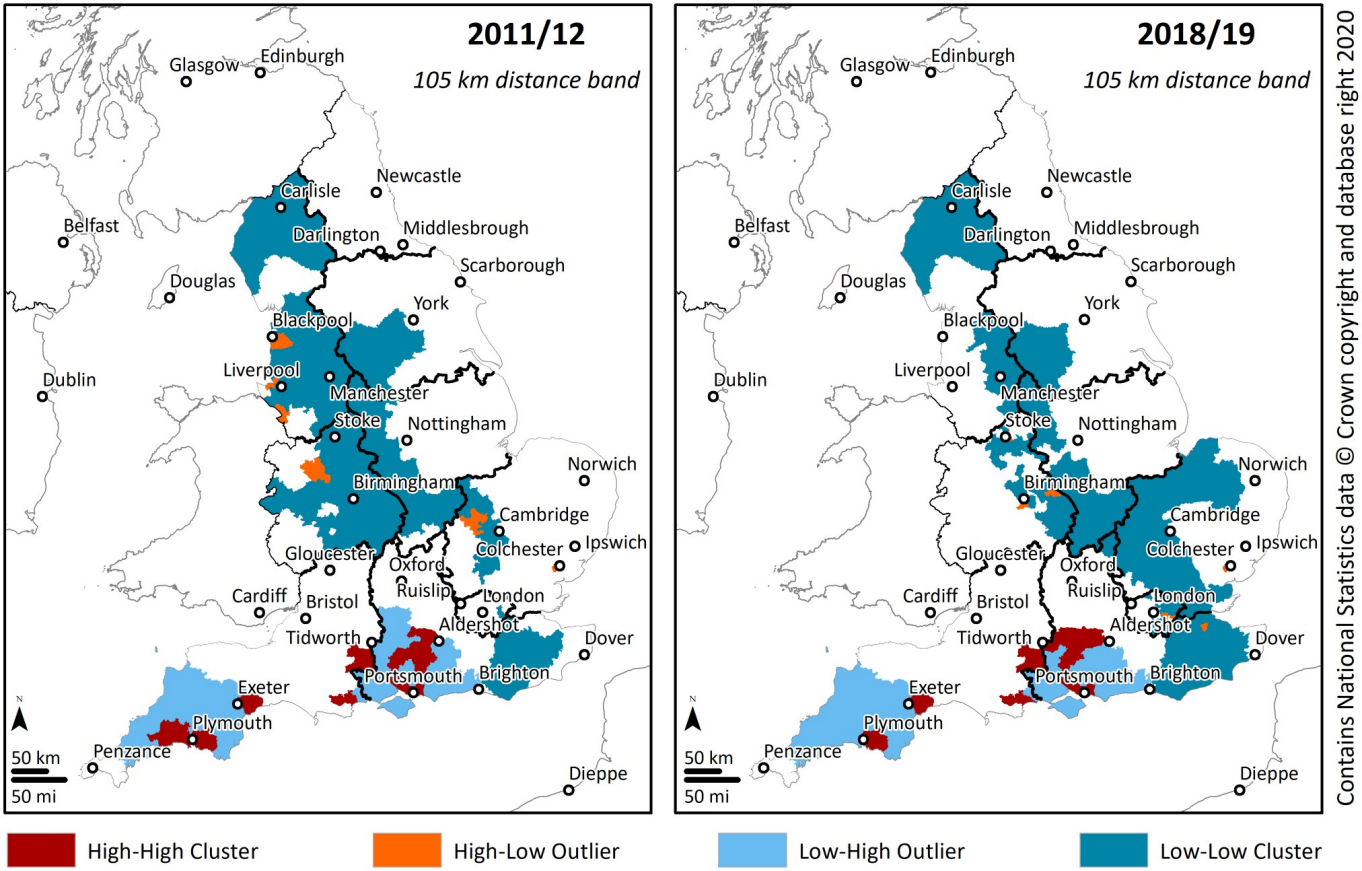
Clusters and outliers of the density of SCPP pupils (105 km distance band).

Clusters of constituencies with high and low density of SCPP pupils, as well as spatial outliers, as assessed by the Anselin local Moran’s I statistic with a fixed distance band of 105 km can be seen in Fig 3.

When considering a distance of 105 km to each constituency, in 2011/12, a total of 22 constituencies were part of a cluster in which the density of SCPP pupils was high as those of its neighbours (HH cluster). In the same period, 187 constituencies were part of a LL cluster, where a constituency and its neighbours had a low density of SCPP pupils. Employing the same distance of 105 km, in 2018/19, these values were effectively retained, 22 constituencies were part of a HH cluster and 189 were part of a LL cluster.

**Fig 4:**
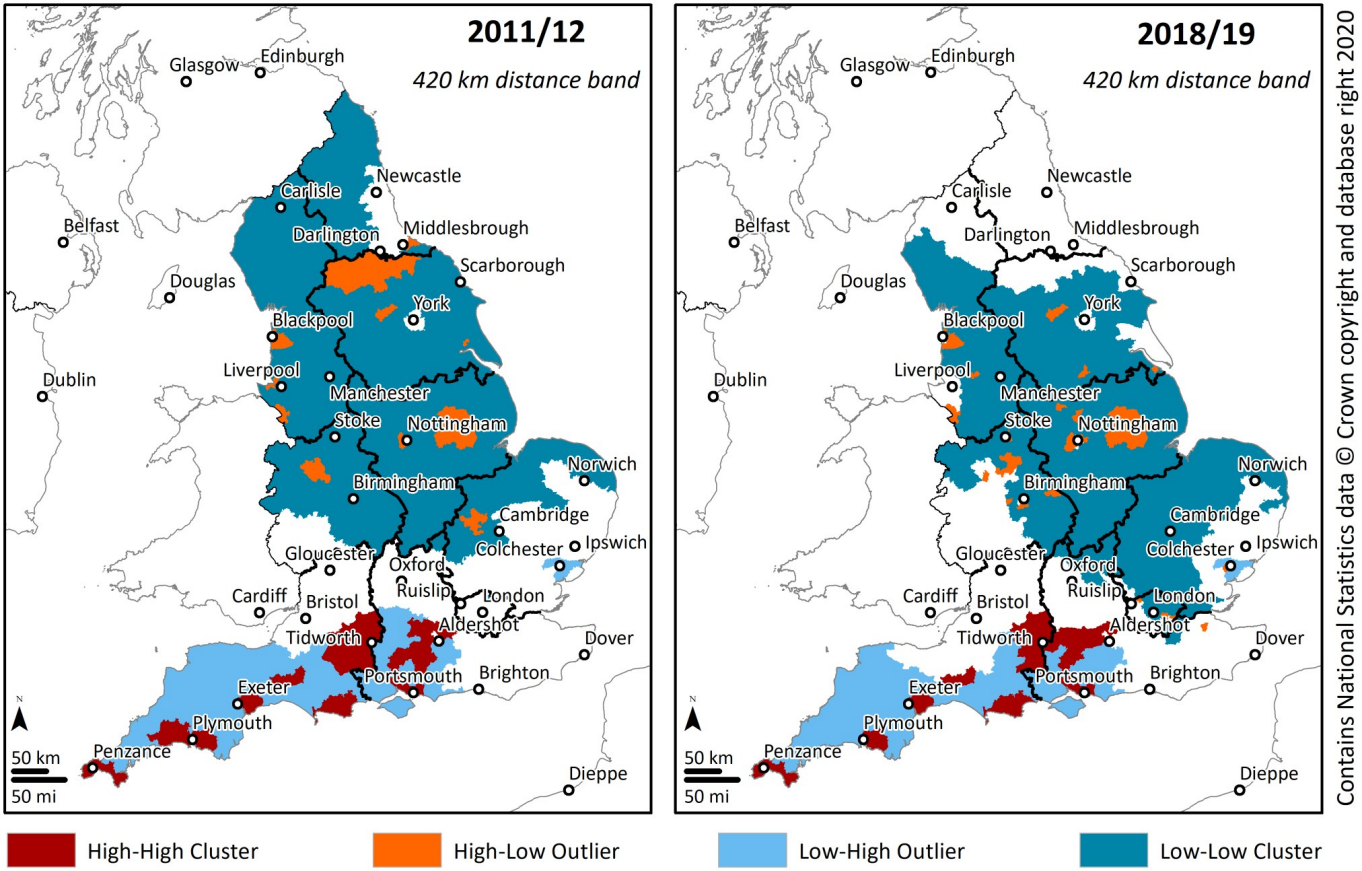
Clusters and outliers of the density of SCPP pupils (420 km distance band).

Shifting the focus to a fixed distance band of 420 km (Fig 4), in 2011/12, a total of 30 constituencies were part of a HH cluster, while 234 constituencies were part of a LL cluster. However, in 2018/19, the values registered a greater change when compared with the calculations done with a distance band of 105 km. Constituencies with a density of SCPP pupils high as those of its neighbours (HH cluster) decreased their count to 28. While constituencies with a density of SCPP pupils low as those of its neighbours increased to 266.

The same pattern could be found when analysis the spatial outliers. Considering a distance band of 105 km, in 2011/12 there were 11 constituencies identified as an HL outlier, where constituencies evidenced high density of SCPP pupils but were surrounded by areas of lower density. In 2018/19, the number remained the same, 11 constituencies.

For the distance band of 420 km, in 2011/2012, the number of constituencies with a high density of SCPP pupils and surrounded by areas of lower density identified as an HL outlier, was 20, and it increased to 31 in 2018/2019. The results show that, when considering a distance of 105 km, despite the sharp increase in the density of SCPP pupils, the clustering pattern remained similar over the study period. On the other hand, the long-distance range registered greater change mostly due to the increase of the area with a high density of SCPP pupils and surrounded by areas of lower density (HL outlier). This is again an indication of dispersion when considering a macro scale.

Overall, the results suggest that the clustering pattern remained identical when considering constituencies that were part of a cluster in which the density of SCPP pupils was high as those of its neighbours (HH cluster). This finding indicates that there are specific high-density constituencies where military families concentrate (e.g., Plymouth, Portsmouth, Tidworth), and this specific pattern did not change during the study period.

**Fig 5:**
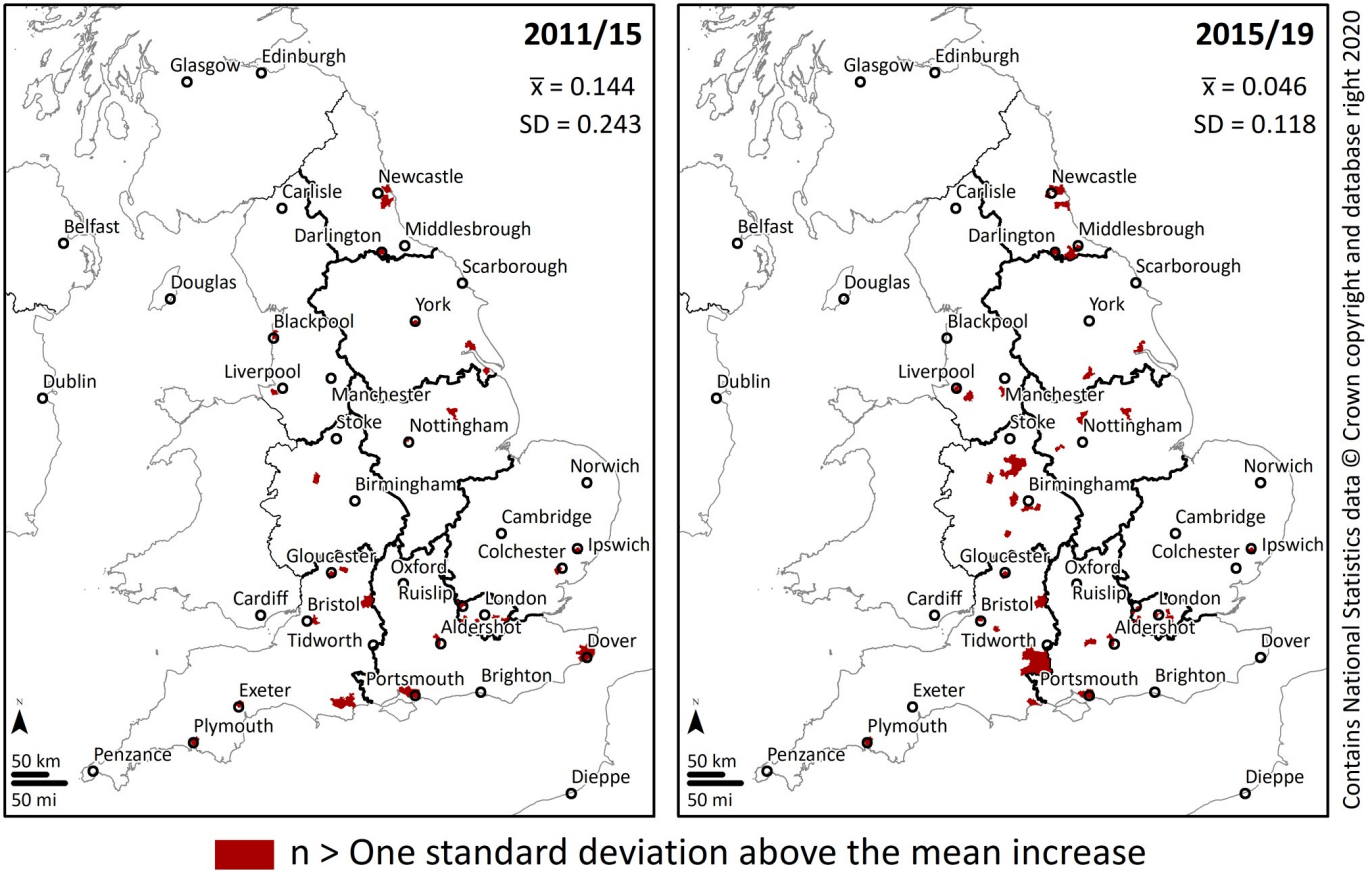
Constituencies with a change in the density of SSP pupils greater than one standard deviation above the mean increase.

Fig 5 shows the location of the areas with the highest change in the density of SCPP pupils in the two halves of the study period. Several of the areas of highest increase were also located in the same areas previously identified as HH cluster, where a constituency and its neighbours had a high density of SCPP pupils (Figs 3 and 4). However, results from Fig 5 also show a high increase in areas outside the high-density core areas. This is more noticeable in the second half of the study period and across the central regions of England.

## 4. Discussion

In England, the distribution of military families is spatially uneven, with varying degrees of concentration giving rise to varying densities. The results of this study show that most of England’s military families with children of school age live in two regions: England’s South West and South East. Less than two percent of the 533 constituencies concentrate one quarter of England’s SCPP pupils. The ten constituencies with the highest densities equate to 0.30% of England’s territory, yet in 2018/19 they concentrated 11% of England’s SCPP pupils. It is argued that a significant cause of this spatial pattern is that the main areas of concentration coincide with areas with a strong presence of military facilities, i.e. the Tidworth Garrison in the South West of England and the Colchester Garrison in the South East. This is not an unexpected finding as it was assumed that most military families would live in or near military establishments and therefore it is argued that the location of military facilities explain why the distribution of military families is spatially uneven.

The findings show that the relocations associated with SDSR 2010 would appear to have had a mensurable impact on the military family’s population in England, proxied through the increasing number of service children. Between 2011 and 2019 the average density of England’s SCPP pupils increased 88%. This dynamic was evident in the measures of central tendency which grew over the study period. However, across England, this increase was far from uniform.

A significant finding relates to the importance of the scale factor. Within a shorter distance (105 km radius), SCPP pupils’ growth followed a spatial pattern that has become increasingly consolidated over the study period. This is probably explained by the movement of personnel from Germany back to the UK, along with the SDSR 2010 impact of also closing military garrisons and bases within the UK. The process would appear to have concentrated, or clustered, military family’s populations around large military bases or garrisons.

However, this increasingly clustered pattern had different phases. When considering a radius of 105 km, findings indicated that over the study period, the increase in SCPP pupils was coupled with a more clustered pattern due to a process of progressive territorial concentration. This study did not seek to explain why but focused on the where, as there is no data that could help explain the commuting patterns of military families within this 105 km radius. However, most of England’s very large Garrisons are located in rural areas such as North Yorkshire, and Wiltshire, and the wider literature would suggest that England’s rural residents tend to have longer commutes than average [39]. Moving to a radius of 420 km, beyond commuting distance, this increase in SCPP pupils was accompanied by a process of growing displacement and scattering at a macro scale.

Still at a macro scale, the results corroborate the same findings when comparing median-to-mean density ratios over the years (Table 2). Between 2011 and 2017 the ratio increased, while between 2017 and 2019 it decreased. This indicates the same two distinct dynamics mentioned previously: 1) between 2011 and 2017 the proportion of SCPP pupils in constituencies with lower densities increased, indicating a more balanced distribution and; 2) between 2017 and 2019 a smaller percentage of SCPP pupils were located in constituencies with lower densities, while a larger percentage were in higher density constituencies, indicating a less balanced distribution of military families. Therefore, between 2011 and 2017 the median-to-mean density ratio evidenced dispersion. Whereas between 2017 and 2019 there was a trend for constituencies with more SCPP pupils to grow in numbers while constituencies with less densities were losing SCPP pupils, evidencing of concentration. Since SDSR closed military facilities within the UK, it could be assumed that, at a macro scale, beyond commuting distance, when the results show an increase of military families coupled with a more dispersed pattern, this pattern cannot be explained by the fragmentation or creation of additional military facilities. If the coming years confirm the trend identified here, the paradigm of military families living on-base with serving personnel is shifting when a macro (country) scale is considered.

Between the beginning and end of the study period, the results indicate a decrease, albeit slightly, in the number of constituencies with a high density and surrounded by other constituencies with an equally high density of SCPP pupils. This occurred despite the continuous growth of the military families’ total, year after year. Again, this finding suggests an underlying dynamic that, at a macro scale, military families have become less clustered. The location of the HH clusters suggests a clear association with large military facilities. However, when analysing density change, several areas stand out that are not located in areas of high density of military families. In fact, several outliers with a high-density increase are apparent in areas that have a low density of military families with dependent children.

This study argues that the implementation of SDSR has coincided with a substantial increase in the growth and spatial distribution of military families with dependent children in England. When considering a short distance from each constituency, this restructuring appears to show a progressive clustering of military families into increasingly fewer military facilities. However, this study also demonstrates that, at a macro scale, families began to disperse beyond a commuting distance threshold.

It is therefore argued that this displacement and scattering at a macro scale has direct implications regarding the support of military families. A good example of the impact that such a dispersal might have on the population is the allocation of accommodation. Accompanied married service personnel, or those in a civil partnership, are eligible to apply for Service Families Accommodation (SFA). SFA housing is provided by the UKMOD so that the serving partner can live as close to their duty station as possible and cohabit with the family [40]. SFA is available on or near military facilities, and a subsidised charge is deducted from the service person salary. The charges are subsidised compared to market rate [41]. Therefore, living on SFA means that significant savings can be made on commuting time, travel costs and housing costs. However, for the minority of families that live in private housing, dispersed and off base, it means that they can choose to become geographically stable. Nonetheless, the choice of becoming geographically stable comes at a cost of not having formal support for housing and not being able to cohabit with the serving parent if the base is beyond commuting distance. In addition, it also reduces the families’ ability to access support which, as identified by the House of Commons Defence Committee [20], is not currently adapted to support families living away from military garrisons or bases. However, it must be noted that the housing issue is something that the FAM [21] seeks to address.

UK research indicates the challenges that military spouses face when looking for employment as part of a mobile population [3], suggesting that these challenges can impact negatively on the psychological well-being of military spouses [42]. This is consistent with US and Canadian literature, where career and employment opportunities of military spouses have been shown to be negatively influenced by frequent and repeated military relocations [43–45]. Military spouses living in SFA cannot commit to being geographically stable, i.e. sooner or later they will have to move with the partner that is serving. Living dispersed and geographically stable, potentially negates difficulties previously experienced in gaining employment as a mobile population.

The results also show that, contrary to the overall population, military families do not tend to live in metropolitan areas. This has potential implications for the resources available in a sparsely populated, more rural area. It does raise the question of how prepared communities and local government were for, what appeared to be, a significant increase in military families in relation to accommodation, school places and transport facilities, and what resources were made available.

It is acknowledged that this study is exploratory and there are always limitations with using proxy measures. It is also acknowledged that only an estimate can be produced for England because SCPP data is not available for Wales, Scotland and Northern Ireland. In addition, there will be variations in military families with dependent children at the sub-constituency level, but a more detailed scale cannot be explored using the current publicly available SCPP data. That said, at this time there is no alternative data available to study the geospatial distribution of military families. It is argued that this approach gives a robust indication of where the military families are located in England and more importantly, it is a method that can be repeated annually to provide government with this vital information, which at this time they have no other means of obtaining.

## 5. Conclusion

This study is empirical evidence of how the implementation of SDSR 2010 has coincided with the significant increase in the number of military families across England. The results show the main areas of concentration of military families, but more importantly, it was also possible to identify how over time this community has begun to disperse, indicating a change in the relationship between the British Armed Forces and serving personnel’s families. This trend, towards a growing displacement and scattering, challenges the existent paradigm for formal and informal support currently available to military families.

We argue that the findings unveil spatial dynamics that have practical issues of housing, employment and education that should be considered to inform decisions related to the development and implementation of targeted policies for military families in the future.

## Supporting information

S1 Data Service pupil premium data used in the study. (XLSX)
